# Supporting Early Scientific Thinking Through Curiosity

**DOI:** 10.3389/fpsyg.2020.01717

**Published:** 2020-08-05

**Authors:** Jamie J. Jirout

**Affiliations:** Curry School of Education and Human Development, University of Virginia, Charlottesville, VA, United States

**Keywords:** curiosity, scientific reasoning, scientific thinking, information seeking, exploration, learning

## Abstract

Curiosity and curiosity-driven questioning are important for developing scientific thinking and more general interest and motivation to pursue scientific questions. Curiosity has been operationalized as preference for uncertainty (Jirout and Klahr, 2012), and engaging in inquiry-an essential part of scientific reasoning-generates high levels of uncertainty (Metz, 2004; van Schijndel et al., 2018). This perspective piece begins by discussing mechanisms through which curiosity can support learning and motivation in science, including motivating information-seeking behaviors, gathering information in response to curiosity, and promoting deeper understanding through connection-making related to addressing information gaps. In the second part of the article, a recent theory of how to promote curiosity in schools is discussed in relation to early childhood science reasoning. Finally, potential directions for research on the development of curiosity and curiosity-driven inquiry in young children are discussed. Although quite a bit is known about the development of children’s question asking specifically, and there are convincing arguments for developing scientific curiosity to promote science reasoning skills, there are many important areas for future research to address how to effectively use curiosity to support science learning.

## Scientific Thinking and Curiosity

Scientific thinking is a type of knowledge seeking involving intentional information seeking, including asking questions, testing hypotheses, making observations, recognizing patterns, and making inferences (Kuhn, 2002; Morris et al., 2012). Much research indicates that children engage in this information-seeking process very early on through questioning behaviors and exploration. In fact, children are quite capable and effective in gathering needed information through their questions, and can reason about the effectiveness of questions, use probabilistic information to guide their questioning, and evaluate who they should question to get information, among other related skills (see Ronfard et al., 2018 for review). Although formal educational contexts typically give students questions to explore or steps to follow to “do science,” young children’s scientific thinking is driven by natural curiosity about the world around them, and the desire to understand it and generate their own questions about the world (Chouinard et al., 2007; Duschl et al., 2007; French et al., 2013; Jirout and Zimmerman, 2015).

### What Does Scientific Curiosity Look Like?

**FIGURE 1:**
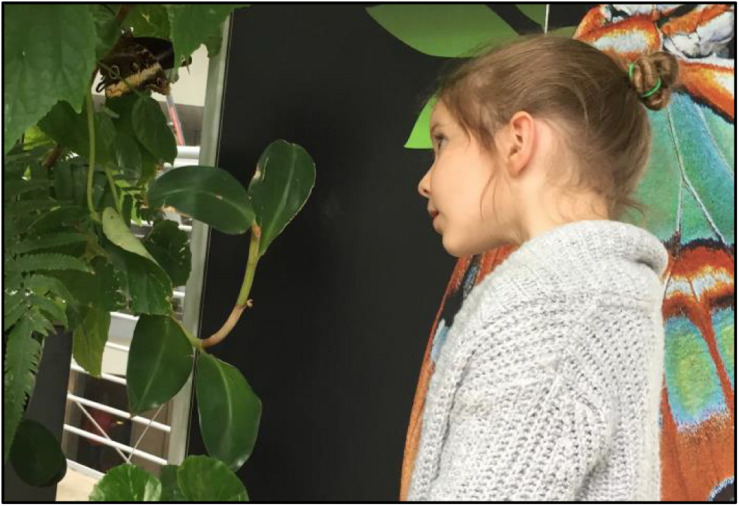
A child looks intently at a butterfly, becoming curious about the many things she wonders based on her observations.

Curiosity is defined here as the desire to seek information to address knowledge gaps resulting from uncertainty or ambiguity (Loewenstein, 1994; Jirout and Klahr, 2012). Curiosity is often seen as ubiquitous within early childhood. Simply observing children can provide numerous examples of the bidirectional link between curiosity and scientific reasoning, such as when curiosity about a phenomenon leads to experimentation, which, in turn, generates new questions and new curiosities. For example, an infant drops a toy to observe what will happen. When an adult stoops to pick it up, the infant becomes curious about how many times an adult will hand it back before losing interest. Or, a child might observe a butterfly over a period of time, and wonder why it had its wings folded or open at different points, how butterflies fly, why different butterflies are different colors, and so on (see Figure 1). Observations lead to theories, which may be immature, incomplete, or even inaccurate, but so are many early scientific theories. Importantly, theories can help identify knowledge gaps, leading to new instances of curiosity and motivating children’s information seeking to acquire new knowledge and, gradually, correct misconceptions. Like adults, children learn from their experiences and observations and use information about the probability of events to revise their theories (Gopnik, 2012).

Although this type of reasoning is especially salient in science, curiosity can manifest in many different types of information seeking in response to uncertainty, and is similar to critical thinking in other domains of knowledge and to active learning and problem solving more generally (Gopnik, 2012; Klahr et al., 2013; Saylor and Ganea, 2018). The development of scientific thinking begins as the senses develop and begin providing information about the world (Inhelder and Piaget, 1958; Gopnik et al., 1999). When they are not actively discouraged, children need no instruction to ask questions and explore, and the information they get often leads to further information seeking. In fact, observational research suggests that children can ask questions at the rate of more than 100 per hour (Chouinard et al., 2007)! Although the adults in a child’s life might tire of what seems like relentless questioning (Turgeon, 2015), even young children can modify their beliefs and learn from the information they receive (Ronfard et al., 2018). More generally, children seek to understand their world through active exploration, especially in response to recognizing a gap in their understanding (Schulz and Bonawitz, 2007). The active choice of what to learn, driven by curiosity, can provide motivation and meaning to information and instill a lasting positive approach to learning in formal educational contexts.

### How Does Curiosity Develop and Support Scientific Thinking?

There are several mechanisms through which children’s curiosity can support the development and persistence of scientific thinking. Three of these are discussed below, in sequence: that curiosity can (1) motivate information-seeking behavior, which leads to (2) question-asking and other information-seeking behaviors, which can (3) activate related previous knowledge and support deeper learning. Although we discuss these as independent, consecutive steps for the sake of clarity, it is much more likely that curiosity, question asking and information seeking, and cognitive processing of information and learning are all interrelated processes that support each other (Oudeyer et al., 2016). For example, information seeking that is not a result of curiosity can lead to new questions, and as previous knowledge is activated it may influence the ways in which a child seeks information.

#### Curiosity as a Motivation for Information Seeking

Young children’s learning is driven by exploration to make sense of the world around them (e.g., Piaget, 1926). This exploration can result from curiosity (Loewenstein, 1994; Jirout and Klahr, 2012) and lead to active engagement in learning (Saylor and Ganea, 2018). In the example given previously, the child sees that some butterflies have open wings and some have closed wings, and may be uncertain about why, leading to more careful observations that provide potential for learning. Several studies demonstrate that the presence of uncertainty or ambiguity leads to higher engagement (Howard-Jones and Demetriou, 2009) and more exploration and information seeking (Berlyne, 1954; Lowry and Johnson, 1981; Loewenstein, 1994; Litman et al., 2005; Jirout and Klahr, 2012). For example, when children are shown ambiguous demonstrations for how a novel toy works, they prefer and play longer with that toy than with a new toy that was demonstrated without ambiguity (Schulz and Bonawitz, 2007). Similar to ambiguity, surprising or unexpected observations can create uncertainty and lead to curiosity-driven questions or explanations through adult–child conversations (Frazier et al., 2009; Danovitch and Mills, 2018; Jipson et al., 2018). This curiosity can promote lasting effects; Shah et al. (2018) show that young children’s curiosity, reported by parents at the start of kindergarten, relates to academic school readiness. In one of the few longitudinal studies including curiosity, research shows that parents’ promotion of curiosity early in childhood leads to science intrinsic motivation years later and science achievement in high school (Gottfried et al., 2016). More generally, curiosity can provide a remedy to boredom, giving children a goal to direct their behavior and the motivation to act on their curiosity (Litman and Silvia, 2006).

#### Curiosity as Support for Directing Information-Seeking Behavior

Gopnik et al. (2015) suggest that adults are efficient in their attention allocation, developed through extensive experience, but this attentional control comes at the cost of missing much of what is going on around them unrelated to their goals. Children have less experience and skill in focusing their attention, and more exploration-oriented goals, resulting in more open-ended exploratory behavior but also more distraction. Curiosity can help focus children’s attention on the specific information being sought (e.g., Legare, 2014). For example, when 7–9-year-old children completed a discovery-learning task in a museum, curiosity was related to more efficient learning-more curious children were quicker and learned more from similar exploration than less-curious children (van Schijndel et al., 2018). Although children are quite capable of using questions to express curiosity and request specific information (Berlyne, 1954; Chin and Osborne, 2010; Jirout and Zimmerman, 2015; Kidd and Hayden, 2015; Luce and Hsi, 2015), these skills can and should be strategically supported, as question asking plays a fundamental role in science and is important to develop (Chouinard et al., 2007; Dewey, 1910; National Governors Association, 2010; American Association for the Advancement of Science [AAAS], 1993; among others). Indeed, the National Resource Council (2012) National Science Education Standards include question asking as the *first* of eight scientific and engineering practices that span all grade levels and content areas.

Children are proficient in requesting information from quite early ages (Ronfard et al., 2018). Yet, there are limitations to children’s question asking; it can be “inefficient.” For example, to identify a target object from an array, young children often ask confirmation questions or make guesses rather than using more efficient “constraint-seeking” questions (Mills et al., 2010; Ruggeri and Lombrozo, 2015). However, this behavior is observed in highly structured problem-solving tasks, during which children likely are not very curious. In fact, if the environment contains other things that children are curious about, it could be more efficient to use a simplistic strategy, freeing up cognitive resources for the true target of their curiosity. More research is needed to better understand children’s use of curiosity-driven questioning behavior as well as exploration, but naturalistic observations show that children do ask questions spontaneously to gain information, and that their questions (and follow-up questions) are effective in obtaining desired information (Nelson et al., 2004; Kelemen et al., 2005; Chouinard et al., 2007).

#### Curiosity as Support for Deeper Learning

Returning to the definition of curiosity as information seeking to address knowledge gaps, becoming curious-by definition-involves the activation of previous knowledge, which enhances learning (VanLehn et al., 1992; Conati and Carenini, 2001). The active learning that results from curiosity-driven information seeking involves meaningful cognitive engagement and constructive processing that can support deeper learning (Bonwell and Eison, 1991; King, 1994; Loyens and Gijbels, 2008). The constructive process of seeking information to generate new thinking or new knowledge in response to curiosity is a more effective means of learning than simply receiving information (Chi and Wylie, 2014). Even if information is simply given to a child as a result of their asking a question, the mere process of recognizing the gap in one’s knowledge to have a question activates relevant previous knowledge and leads to more effective storage of the new information within a meaningful mental representation; the generation of the question is a constructive process in itself. Further, learning more about a topic allows children to better recognize their related knowledge and information gaps (Danovitch et al., 2019). This metacognitive reasoning supports learning through the processes of activating, integrating, and inferring involved in the constructive nature of curiosity-drive information seeking (Chi and Wylie, 2014). Consistent with this theory, Lamnina and Chase (2019) showed that higher curiosity, which increased with the amount of uncertainty in a task, related to greater transfer of middle school students’ learning about specific science topics.

## Promoting Curiosity in Young Children

Curiosity is rated by early childhood educators as “very important” or “essential” for school readiness and considered to be even more important than discrete academic skills like counting and knowing the alphabet (Heaviside et al., 1993; West et al., 1993), behind only physical health and communication skills in importance (Harradine and Clifford, 1996). Engel (2011, 2013) finds that curiosity declines with development and suggests that understanding how to promote or at least sustain it is important. Although children’s curiosity is considered a natural characteristic that is present at birth, interactions with and responses from others can likely influence curiosity, both at a specific moment and context and as a more stable disposition (Jirout et al., 2018). For example, previous work suggests that curiosity can be promoted by encouraging children to feel comfortable with and explore uncertainty (Jirout et al., 2018); experiences that create uncertainty lead to higher levels of curious behavior (e.g., Bonawitz et al., 2011; Engel and Labella, 2011; Gordon et al., 2015).

One strategy for promoting curiosity is through classroom climate; children should feel safe and be encouraged to be curious and exploration and questions should be valued (Pianta et al., 2008). This is accomplished by de-emphasizing being “right” or all-knowing, and instead embracing uncertainty and gaps in one’s own knowledge as opportunities to learn. Another strategy to promote curiosity is to provide support for the information-seeking behaviors that children use to act on their curiosity. There are several specific strategies that may promote children’s curiosity (see Jirout et al., 2018, for additional strategies), including:

1. Encourage and provide opportunities for children to explore and “figure out,” emphasizing the value of the process (exploration) over the outcome (new knowledge or skills). Children cannot explore if opportunities are not provided to them, and they will not ask questions if they do not feel that their questions are welcomed. Even if opportunities and encouragement are provided, the fear of being wrong can keep children from trying to learn new things (Martin and Marsh, 2003; Martin, 2011). Active efforts to discover or “figure out” are more effective at supporting learning than simply telling children something or having them practice learned procedures (Schwartz and Martin, 2004). Children can explore when they have guidance and support to engage in think-aloud problem solving, instead of being told what to try or getting questions answered directly (Chi et al., 1994).
2. Model curiosity for children, allowing them to see that others have things that they do not know and want to learn about, and that others also enjoy information-seeking activities like asking questions and researching information. Technology makes information seeking easier than it has ever been. For example, children are growing up surrounded by internet-connected devices (more than 8 per capita in 2018), and asking questions is reported to be one of the most frequent uses of smart speakers (NPR-Edison Research Spring, 2019). Observing others seeking information as a normal routine can encourage children’s own question asking (McDonald, 1992).
3. Children spontaneously ask questions, but adults can encourage deeper questioning by using explicit prompts and then supporting children to generate questions (King, 1994; Rosenshine et al., 1996). This is different from asking “Do you have any questions?,” which may elicit a simple “yes” or “no” response from the child. Instead, asking, “What questions do you have?” is more likely to provide a cue for children to practice analyzing what they do not know and generating questions. The ability to evaluate one’s knowledge develops through practice, and scaffolding this process by helping children recognize questions to ask can effectively support development (Kuhn and Pearsall, 2000; Chin and Brown, 2002).
4. Other methods to encourage curiosity include promoting and reinforcing children’s thinking about alternative ideas, which could also support creativity. Part of being curious is recognizing questions that can be asked, and if children understand that there are often multiple solutions or ways to do something they will be more likely to explore to learn “*how* we know and *why* we believe; e.g., to expose science as a way of knowing” (Duschl and Osborne, 2002, p. 40). Children who learn to “think outside the box” will question what they and others know and better understand the dynamic nature of knowledge, supporting a curious mindset (Duschl and Osborne, 2002).

Although positive interactions can promote and sustain curiosity in young children, curiosity can also be suppressed or discouraged through interactions that emphasize performance or a focus on explicit instruction (Martin and Marsh, 2003; Martin, 2011; Hulme et al., 2013). Performance goals, which are goals that are focused on demonstrating the attainment of a skill, can lead to lower curiosity to avoid distraction or risk to achieving the goal (Hulme et al., 2013). Mastery goals, which focus on understanding and the learning process, support learning for its own sake (Ames, 1993). When children are older and attend school, they experience expectations that prioritize performance metrics over academic and intellectual exploration, such as through tests and state-standardized assessments, which discourages curiosity (Engel, 2011; Jirout et al., 2018). In my own recent research, we observed a positive association between teachers’ use of mastery-focused language and their use of curiosity-promoting instructional practices in preschool math and science lessons (Jirout and Vitiello, 2019). Among 5th graders, student ratings of teacher emphasis on standardized testing was associated with lower observed curiosity-promotion by teachers (Jirout and Vitiello, 2019). It is likely that learning orientations influence children’s curiosity even before children begin formal schooling, and de-emphasizing performance is a way to support curiosity.

In summary, focusing on the process of “figuring out” something children do not know, modeling and explicitly prompting exploration and question asking, and supporting metacognitive and creative thinking are all ways to promote curiosity and support effective cognitive engagement during learning. These methods are consistent with inquiry-based and active learning, which both are grounded in constructivism and information gaps similar to the current operationalization of curiosity (Jirout and Klahr, 2012; Saylor and Ganea, 2018; van Schijndel et al., 2018). Emphasizing performance, such as academic climates focused on teaching rote procedures and doing things the “correct” way to get the right answer, can suppress or discourage curiosity. Instead, creating a supportive learning climate and responding positively to curiosity are likely to further reinforce children’s information seeking, and to sustain their curiosity so that it can support scientific thinking and learning.

## Conclusion: a Call for Research

In this article, I describe evidence from the limited existing research showing that curiosity is important and relates to science learning, and I suggest several mechanisms through which curiosity can support science learning. The general perspective presented here is that science learning can and should be supported by promoting curiosity, and I provide suggestions for promoting (and avoiding the suppression of) curiosity in early childhood. However, much more research is needed to address the complex challenge of educational applications of this work. Specifically, the suggested mechanisms through which curiosity promotes learning need to be studied to tease apart questions of directionality, the influence of related factors such as interest, the impact of context and learning domain on these relations, and the role of individual differences. Both the influence of curiosity on learning and effective ways to promote it likely change in interesting and important ways across development, and research is needed to understand this development-especially through studying change in individuals over time. Finally, it is important to acknowledge that learning does not happen in isolation, and one’s culture and environment have important roles in shaping one’s development. Thus, application of research on curiosity and science learning must include studies of the influence of social factors such as socioeconomic status and contexts, the influence of peers, teachers, parents, and others in children’s environments, and the many ways that culture may play a role, both in the broad values and beliefs instilled in children and the adults interacting with them, and in the influences of behavior expectations and norms. For example, parents across cultures might respond differently to children’s questions, so cross-cultural differences in questions likely indicate something other than differences in curiosity (Ünlütabak et al., 2019). Although curiosity likely promotes science learning across cultures and contexts, the ways in which it does so and effective methods of promoting it may differ, which is an important area for future research to explore. Despite the benefits I present, curiosity seems to be rare or even absent from formal learning contexts (Engel, 2013), even as children show curiosity about things outside of school (Post and Walma van der Molen, 2018). Efforts to promote science learning should focus on the exciting potential for curiosity in supporting children’s learning, as promoting young children’s curiosity in science can start children on a positive trajectory for later learning.

## Ethics Statement

Written informed consent was obtained from the individual(s) and/or minor(s)’ legal guardian/next of kin publication of any potentially identifiable images or data included in this article.

## Author Contributions

JJ conceived of the manuscript topic and wrote the manuscript.

## Conflict of Interest

The author declares that the research was conducted in the absence of any commercial or financial relationships that could be construed as a potential conflict of interest.
